# Multidrug-Resistant (MDR) *Klebsiella variicola* Strains Isolated in a Brazilian Hospital Belong to New Clones

**DOI:** 10.3389/fmicb.2021.604031

**Published:** 2021-04-16

**Authors:** Tatiana Amabile de Campos, Felipe Marques de Almeida, Ana Paula Cardoso de Almeida, Rafael Nakamura-Silva, Mariana Oliveira-Silva, Isabela Felix Alencar de Sousa, Louise Cerdeira, Nilton Lincopan, Georgios Joannis Pappas, André Pitondo-Silva

**Affiliations:** ^1^Departamento de Biologia Celular, Instituto de Ciências Biológicas, Universidade de Brasília, Brasília, Brazil; ^2^Programa de Pós-graduação em Biologia Microbiana, Universidade de Brasília, Brasília, Brazil; ^3^Programa de Pós-graduação em Biologia Molecular, Universidade de Brasília, Brasília, Brazil; ^4^Programa de Pós-graduação em Tecnologia Ambiental, Universidade de Ribeirão Preto, Ribeirão Preto, Brazil; ^5^Instituto de Ciências Biológicas, Universidade de São Paulo, São Paulo, Brazil; ^6^Department of Infectious Diseases, Central Clinical School, Monash University, Melbourne, VIC, Australia; ^7^Programa de Pós-graduação em Odontologia, Universidade de Ribeirão Preto, Ribeirão Preto, Brazil

**Keywords:** *Klebsiella variicola*, antimicrobial resistance, whole genome sequence, pathogenicity, brazilian clones, multidrug-resistant

## Abstract

*Klebsiella variicola* is mainly associated with opportunistic infections and frequently identified as *Klebsiella pneumoniae.* This misidentification implies a wrong epidemiology result as well as incorrect attribution to *K. pneumoniae* as the etiology of some severe infections. Recently, huge efforts have been made to study *K. variicola*, however, the biological aspects of this species are still unclear. Here we characterized five *K. variicola* strains initially identified as *K. pneumoniae*, with a Vitek-2 System and 16S rRNA sequencing. One-step multiplex polymerase chain reaction and Whole Genome Sequencing (WGS) identified them as *K. variicola.* Additionally, WGS analysis showed that all the strains are closely related with *K. variicola* genomes, forming a clustered group, apart from *K. pneumoniae* and *K. quasipneumoniae.* Multilocus sequence typing analysis showed four different sequence types (STs) among the strains and for two of them (Kv97 and Kv104) the same ST was assigned. All strains were multidrug-resistant (MDR) and three showed virulence phenotypes including invasion capacity to epithelial cells, and survival in human blood and serum. These results showed the emergence of new *K. variicola* clones with pathogenic potential to colonize and cause infection in different tissues. These characteristics associated with MDR strains raise great concern for human health.

## Introduction

*Klebsiella variicola* is a Gram-negative, facultative anaerobic and non-motile bacillus belonging to *Enterobacterales*. The species is mainly associated with opportunistic infections, such as those of the bloodstream (BSI) (Maatallah et al., 2014), urinary tract (Potter et al., 2018), respiratory tract (Rodríguez-Medina et al., 2019), and neonatal outbreaks (Piepenbrock et al., 2020). When associated with BSI, the infections presented high mortality rates, but the strains did not show known virulence factors such as capsular types or mucoid phenotype (Maatallah et al., 2014). Although it has an opportunistic nature, hypervirulent strains were described in outbreaks in Bangladesh (Farzana et al., 2019) and China (Lu et al., 2018). The association among hypervirulence and multidrug resistance becomes a more worrying scenario for the public healthcare system.

*K. variicola* are frequently misidentified as *K. pneumoniae* by conventional biochemical tests and automated microbiology systems used worldwide (Rodríguez-Medina et al., 2019). Nevertheless, genomic information surveys have recently demonstrated that this species is also associated with severe human infections (Fonseca et al., 2017; Barrios-Camacho et al., 2019; Srinivasan and Rajamohan, 2020). Although not widely studied as *K. pneumoniae*, there are reports indicating that *K. variicola* shows clear differences related to infection, highlighting the importance of its early diagnosis (Rodríguez-Medina et al., 2019). In this report we characterized five *K. variicola* strains (Kv15, Kv35, Kv57, Kv97, and Kv104), first identified as *K. pnenumoniae*. All these strains were submitted to genomic characterization to determine their sequence types (STs), virulence, and antimicrobial resistance (AMR) genes. Biological assays such as toxicity and invasion to epithelial cells, hypermucosviscousity and survival in human blood and serum were performed to evaluate their pathogenic potential.

## Materials and Methods

### Bacterial Strains Isolation, Growth Conditions, and Storage

The five *K. variicola* strains included in the present study, named Kv15, Kv35, Kv57, Kv97, and Kv104, were isolated at the University Hospital of Brasília, the tertiary hospital at the University of Brasília, located in Brasília in the Brazilian Federal District (DF), between September 2013 and December 2017. The strains were initially identified as *K. pneumoniae* by a VITEK 2 system (BioMérieux) and maintained at −80°C in 15% glycerol.

Thereafter, the bacterial identification was confirmed by molecular methods. Initially, the isolates were typed by 16S rRNA sequencing (Weisburg et al., 1991). After that, *K. variicola* identification was confirmed by means of the one-step multiplex polymerase chain reaction (PCR) protocol proposed by Fonseca et al. (2017). All *K. variicola* identified were used for biological and genomic characterization.

### Antimicrobial Susceptibility Profile

Antimicrobial susceptibility testing was performed by the disk diffusion method as recommended by the Clinical and Laboratory Standards Institute (CLSI) (2020). All antibiotics recommended for *Enterobacterales* were tested, considering the particularities for *Klebsiella* spp. For this approach 37 different antibiotic disks (Oxoid Ltd., Basingstoke, United Kingdom) were used (Supplementary Material 1). Each strain was considered susceptible or non-susceptible (either intermediate or resistant) to each antibiotic tested. Based on the susceptibility profile, each strain was classified into different categories including, multidrug-resistant (MDR), extensively drug-resistant (XDR) and pandrug-resistant (PDR) (Magiorakos et al., 2012).

### Biofilm Production Assays

A volume of 100 μL from an optical density at 600 nm (OD_60__0 *n*__*m*_) culture of each strain, previously grown in Luria-Bertani (LB) broth (Sigma Aldrich) at 37°C, were transferred to a well of a 24 well micro-plate containing 100 μL of LB. After 24 h of incubation at 37°C, the supernatant was discharged and the plates were washed twice with NaCl 0.9%. The pellets were fixed with methanol (15 min at room temperature), dried, and stained with violet crystal. After the solubilization with acetic acid 33%, the OD_57__0 *n*__*m*_ absorbance from each well was read using a Spectramax (Promega). The assay was performed in triplicate for all strains and for the negative control (Stepanović et al., 2004).

### Hypermucosviscosity

The hypermucoviscous (Hmv) phenotype was investigated by a string test (Mazloum et al., 2016). The strains were inoculated on Mueller–Hinton agar (Oxoid) and incubated for 24 h at 37°C. Following bacterial growth, a bacteriological loop was used to touch and lift vertically an isolated colony. Formation of a mucus filament of > 5 mm was considered positive for hypermucoviscosity. All the strains were tested in triplicate.

### Bacterial Human Blood and Serum Survival

Bacterial suspensions (10^7^ bacterial cells) obtained from a culture of 18 h grown at 37°C in LB broth were added in 550 μL of human blood and in 550 μL human serum and incubated for 0, 30, 60, and 120 min at 37°C. Thereafter, aliquots of 10 μL from each inoculum were plated in MacConkey Agar (Oxoid Ltd.) and the plates were incubated at 37°C for 18 h for colony forming unit (CFU) recovery quantification (DeLeo et al., 2017). For the assays, blood and serum were collected from five healthy donors. All strains were tested in triplicate for each donor.

### Bacterial Invasion and Toxicity to HEp-2 Epithelial Cells

Bacterial invasion to epithelial cells and cytotoxicity were evaluated with HEp-2 cells by using multiplicity of infection 1:1,000 (HEp-2 cells: bacterial strains) (Favre-Bonte et al., 1999). Following the infection, the HEp-2 cells were incubated at 37°C and 5% CO_2_. After 30 min the cells were washed with phosphate-buffered saline (PBS) and the medium was replaced by Dulbecco’s Modified Eagle’s medium (Gibco) with 10 mg/mL of gentamicin (Sigma Aldrich). Bacterial invasion and intracellular survival were evaluated by CFU quantification at 24 h post infection (h.p.i.). Bacterial cytotoxicity was determined by MTT (3-4, 5-dimethylthiazolyl-2)-2, 5-diphenyltetrazolium bromide assays (Sigma Aldrich) at 3 h.p.i (Campos et al., 2018). All assays were carried out in triplicate for all strains.

### Genome Sequencing

Genomic DNA samples were sequenced on an Illumina MiSeq platform (Illumina Inc., San Diego, CA) using 250-bp paired-end reads for the strains Kv15, Kv97, and Kv104 and on an Illumina NextSeq platform (Illumina Inc., San Diego, CA), using 75-bp paired-end reads for the strains Kv35 and Kv57.

### Genome Assembly and Annotation

*De novo* genome assembly was performed with Unicycler (Wick et al., 2017) v0.4.8 using the MpGAP pipeline v1.0^1^ with default parameters. Genome completeness was assessed with BUSCO v4.1.0, using the enterobacterales_odb10 dataset (Seppey et al., 2019).

Genome annotation was performed with the bacannot pipeline v1.0^2^ using 85% gene coverage and identity thresholds for virulence gene annotations. Through this pipeline, AMR genes were detected with CARD-RGI database (version 3.0.7) (Jia et al., 2017) and AMRFinderPlus v3.2.1 (Feldgarden et al., 2019) tools, while VFDB (Liu et al., 2019) was used to identify virulence factors (accessed in November 2019). Additionally, AMR genes were also predicted using ResFinder 4.0 (Bortolaia et al., 2020). Capsule synthesis (K) and lipopolysaccharide (O) loci were identified using Kaptive (Wick et al., 2018). Multilocus sequence typing (MLST) was performed with *K. variicola* MLST scheme (accessed on 1th September 2019) (Barrios-Camacho et al., 2019). Plasmid replicons were detected with Plasmidfinder v2.1 (Carattoli et al., 2014).

### Comparative Genomics

Genome-based taxonomy analysis was performed with the Type Strain Genome Server (TYGS) (Meier-Kolthoff and Göker, 2019). Average Nucleotide Identity among the five genomes were calculated with fastANI v1.3 (Jain et al., 2018b) and whole genome homology maps were produced with Mashmap (Jain et al., 2018a). The genomes of the five strains were aligned with snippy v4.6.0^3^, using default parameters, to detect single nucleotide polymorphism among them.

For species-level comparative analysis, all the *Klebsiella variicola* complete genomes available in NCBI Refseq in December 2019 were downloaded via ncbi-genome-download tool^4^, totalizing 296 genomes.

With these genomes, an alignment-free genomic distance matrix among them was constructed with Dashing v0.5-7-g841b (Baker and Langmead, 2019) with default parameters. The estimated genomic distances generated by Dashing were in turn projected into a two-dimensional space using multidimensional scaling analysis (MDS), by means of custom-made R language scripts. *K. variicola* pangenome was estimated using PPanGGOLIN v1.1.72 (Gautreau et al., 2020) with default parameters. This pangenome was further used to align and create a gene presence/absence matrix of genes of interest (such as the presence of virulence genes) using PPanGGOLiN align module.

All the MLST profiles deposited in the *K. variicola* MLST database^5^ were downloaded in October 2020 and were used to reconstruct the genetic relationships among them using a minimum spanning tree algorithm (MSTree V2) implemented in GrapeTree v2.2 (Zhou et al., 2018).

### Statistical Analysis

Statistical analysis was performed by using GraphPadPrism v. 8.0 (*GraphPad* Software, San Diego California United States). ANOVA one-way was used to determine the bacterial invasion capacities and intracellular survival. The *t*-test was used for bacterial toxicity for HEp-2 cells determination.

## Results

### Bacterial Strains Isolation and Identification

The strains studied in this work were obtained from a *K. pneumoniae* collection containing 104 isolates from different patients and sources from September 2013 to January 2017. Among all isolates, five were later identified as *K. variicola* (Table 1), corresponding to 4.8% of prevalence within this allegedly *K. pneumoniae* collection. For *K variicola* identification, we used three different techniques: Vitek2 (BioMérieux), 16S rRNA sequencing, and one-step multiplex PCR protocol. Vitek2 identified the strains as *K. pneumoniae*, whereas the 16S sequencing showed 100% identity to both *K. variicola* and *K. pneumoniae*. Conversely, the multiplex PCR approach identified all the strains as *K. variicola*, showing its adequacy to differentiate the two species in this context.

**TABLE 1:** Isolation data, biofilm production, hypermucousviscous phenotype and antimicrobial resistance profile of *Klebsiella variicola* strains.

Strain ID	Culture	Patient data		Isolation period (year-month)	Hmv	Biofilm production	Antimicrobial resistance profile*
		Genera	Birth date (year-month)				
Kv15	Urine	F	1942/Aug	2015/Apr	−	STR	MDR (tobramycin, doxycycline, nitrofurantoin, tetracycline, sulfonamide, trimethoprim + sulfamethoxazole, trimethoprim, nalidixic acid)
Kv35	Blood	F	1973/Sept	2015/Sept	−	W	MDR (ciprofloxacin, tobramycin, cefuroxime, nitrofurantoin, sulphonamide, trimethoprim + sulfamethoxazole, trimethoprim, nalidixic acid)
Kv57	Catheter	M	1961/Sept	2016/Mar	−	M	MDR (tobramycin, doxycycline, nitrofurantoin, sulphonamide, trimethoprim + sulfamethoxazole, trimethoprim)
Kv97	Urine	F	1940/Mar	2017/Jan	+	STR	MDR (ciprofloxacin, tobramycin, nitrofurantoin, sulphonamide, trimethoprim + sulfamethoxazole, trimethoprim)
Kv104	Blood	F	1959/Aug	2017/Jan	+	STR	MDR (ciprofloxacin, tobramycin, nitrofurantoin, sulphonamide, trimethoprim + sulfamethoxazole, trimethoprim)

F, Female; M, Male; Mar, Mach; Aug, August; Sept, September; Hmv, hypermucousviscous phenotype; ST, sequence type; STR, Strong biofilm production M, Moderate biofilm production; W, Weak biofilm production; S, Susceptible to all antimicrobials tested; R, Not Susceptible to up to two antimicrobial categories; MDR, non-susceptible to one or more agents in more the three antimicrobial categories; * all strains are intrinsically resistant to ampicillin.

### Antimicrobial Susceptibility and Biological Assays

**FIGURE 1:**
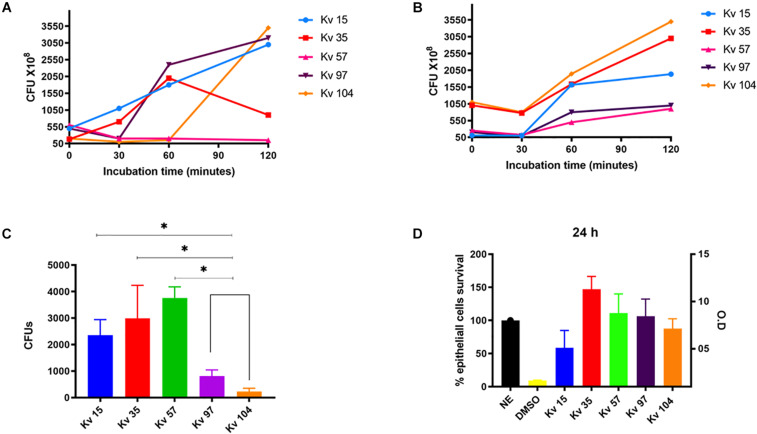
Biological assays used to determine the pathogenic potential of *Klebsiella variicola* strains. **(A)** Bacterial survival after incubation in human blood for different times at 37°C. **(B)** Bacterial survival after incubation in human serum for different times at 37°C. **(C)** Bacterial invasion in HEp-2 cells. **(D)** Bacterial cytotoxicity to HEp-2 cells. The bacterial survival was determined by CFU counting of strains plated from the incubation in blood and serum. Bacterial invasion was determined by CFU counting of strains plated after HEp-2 lysis. Cytotoxicity to HEp-2 cells were determined by the Griss Method. All assays were performed in triplicate. ANOVA one-way were used for statical analysis, *p < 0.05 values were considered significant.

Disk diffusion test showed that all strains were classified as multidrug resistant (MDR), as they were non-susceptible to three different antimicrobial classes (Table 1 and Supplementary Material 1). *In vitro* assays were performed to determine hypermucoviscous phenotype, biofilm production, serum and blood bacterial survival, epithelial cells invasion and citoxicity (Table 1 and Figure 1). Overall, the strains presented capacity to survive and grow after 60 min of incubation in human blood and serum (Figures 1A,B). The strain Kv57, isolated from a catheter culture of a 55 year old man, did not show Hmv phenotype, but presented survival and growth in human serum, as well as high invasion capacity in epithelial cells and high cytotoxicity to HEp-2 cells (reducing 75% of cell viability) (Table 1 and Figure 1). Kv97 and Kv104, isolated from urine culture from elderly female patients, presented Hmv phenotype and strong biofilm production (Table 1). However, both strains showed low capacity to invade Hep2 cells when compared to the others (Figure 1C).

### Genomic Analyses

Sequence data obtained by short-read DNA sequencing was used to assemble the genomes of the five studied strains. The resulting genome assemblies averaged 5.5 Mb and showed high levels (>95%) of gene space completeness based on BUSCO metrics (Supplementary Table 1). Aside from the chromosomal scaffold, the IncFIB(K) plasmid replicon was identified in the genomes of Kv35, Kv97, and Kv104 (Supplementary Table 1). All the genomes have been classified as *K. variicola* by the Type Strain Genome Server (TYGS).

### Molecular Typing

The identification of K and O serotypes is important for rapid detection of outbreaks or high-risk clones, given their links to increased virulence, as is the case of K1 and O1 serotypes (Hsieh et al., 2012; Fang et al., 2016; Patro and Rathinavelan, 2019). Genome analysis enabled the detection of four different capsular synthesis loci (KL60, KL71, KL111, and KL20) and one lipopolysaccharide locus shared among the genomes (O3) (Table 2). In *K. pneumoniae*, the O3 strains is one of the most common serotypes, after O1 and O2.

**TABLE 2:** Antimicrobial resistance genes (ARG), virulence genes (VG), and molecular typing of *Klebsiella variicola* strains.

Strain ID	ARG	K	O	VG	ST
					
Kv15	*oqx*AB, *fos*A, *bla*_LEN_-_9_, *amp*H, *acr*AB	KL60	O3	Capsule, enterobactin, type 1 fimbriae, type3 fimbriae, *acr*R, *omp*K36, *omp*K37	170
Kv35	*oqx*AB, *fos*A, *bla*_LEN__–__2_, *amp*H, *acr*AB	KL71	O3	Capsule, enterobactin, Type 1 fimbriae, Type3 fimbriae, *acr*R, *omp*K36, *omp*K37	171
Kv57	*oqx*AB, *fos*A, *bla*_LEN__–__2_, *amp*H, *acr*AB	KL111	O3	Capsule, enterobactin, type 1 fimbriae, type3 fimbriae, *acr*R, *omp*K36, *omp*K37	168
Kv97	*oqx*AB, *fos*A, *bla*_LEN__12_, *amp*H, *acr*AB	KL20	O3	Capsule, enterobactin, type 1 fimbriae, type3 fimbriae, *acr*R, *omp*K36, *omp*K37	172
Kv104	*oqx*AB, *fos*A, *bla*_LEN__12_, *amp*H, *acr*AB	KL20	O3	Capsule, enterobactin, type 1 fimbriae, typ3 fimbriae, *acr*R, *omp*K36, *omp*K37	172

**K, capsular antigen type; O, O antigen type; ST, sequence type.**

**FIGURE 2:**
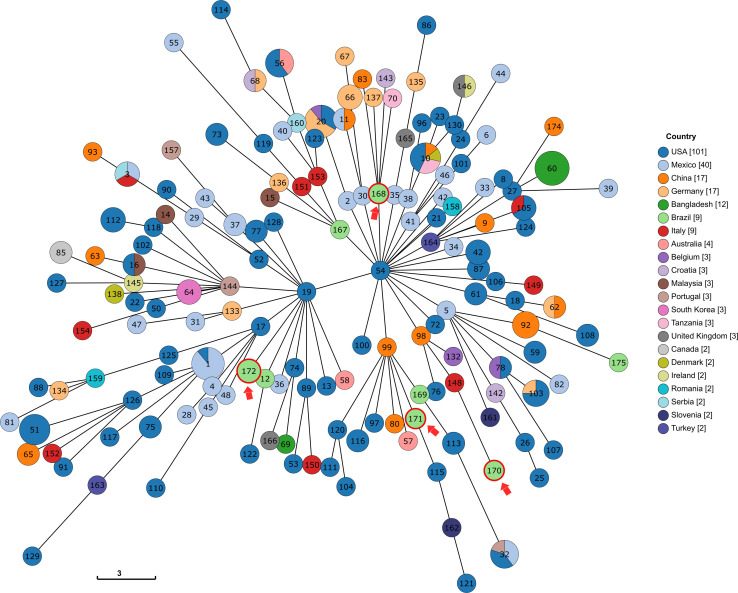
Tree representation of the genetic relationships among the different profiles of the *K. variicola* MLST scheme, produced via GrapeTree with the minimum spanning tree algorithm (MSTree V2). The STs identified in this study are shown with a red arrow. For readability purposes, all the genomes that were the only representant of a country were removed.

Using the INSP MLST scheme for *K. variicola*, four new STs were identified (ST168, ST170, ST171, and ST172), with Kv97 and Kv104 assigned as ST172 (Table 2). The genetic relationships among all known *K. variicola* STs was visualized with GrapeTree, which indicated that the species can be divided into at least two clonal groups (CGs) that seem to originate from STs 19 and 54 (Figure 2). This analysis shows that the studied strains were allocated in both CGs. Interestingly, the strains Kv97 and Kv104, which are the less invasive to Hep-2 cells (Figure 1C and Table 1) have been placed in the CG with ST19 (Figure 2) as a founder whereas the other strains (Kv15, Kv35, and Kv57) were grouped in the other CG with ST54 as a founder (Figure 2). This observation suggests that these CGs may represent populations of *K. variicola* with different phenotypes and gene content dynamics similar to *K. pneumoniae* (Wyres et al., 2019, 2020). Moreover, all the strains from Brazil, including the strains of this study, have been spread in different branches of the tree, highlighting the clonal diversity in Brazil that still needs to be measured.

### Antimicrobial Resistance (AMR) Genes and Virulence Genes (VG)

To support the experimental observation that all analyzed strains are classified as MDR (Table 1), genomic sequences were used to search for AMR genes using three software tools and databases, namely AMRFinderPlus, CARD and Resfinder. The combined results showed the presence of genes related to intrinsic beta-lactam resistance (*amp*H, *bla*_LEN__–__2_, *bla*_LEN__–__9_, *bla*_LEN__–__12_) and fosfomycin (*fos*A) in all strains (Table 2). Moreover, the efflux pump *oqxAB* was also found and has been implicated in low to intermediate resistance to quinoxalines, quinolones, tigecycline, nitrofurantoin, several detergents and disinfectants. In addition, Resfinder identified mutations in the genes *acr*R, *omp*K36, and *omp*K37, that can possibly confer resistance to fluoroquinolones, cephalosporins, and carbapenem, however, further investigation of these mutations is still necessary. The efflux pump *acr*AB was also found among all strains. When overexpressed, this efflux pump contributes to multidrug resistance. Besides, *acr*AB is a resistance factor widespread in *K. pneumoniae* and can to contribute to virulence by providing resistance to host-derived antimicrobial peptides (Swick et al., 2011; Table 2).

The search for virulence genes identified that the strains harbored classical *K. pneumoniae* virulence factors related to the phenolate siderophore enterobactin, type 1 fimbriae, and the *E. coli* common pilus (ECP) locus (Table 2), an adhesive structure produced by all *E. coli* pathogroups that is also produced by *Klebsiella pneumoniae* strains (Alcántar-Curiel et al., 2013). Type 3 fimbriae *mrk*ABCDF and *mrk*HIJ gene loci, which mediate bacterial adhesion and biofilm formation in several surface structures (Wilksch et al., 2011), were also detected in the genomes. Moreover, all strains harbored multiple genes from the type VI secretion system (T6SS), an apparatus involved in bacterial competition, cell invasion, and *in vivo* colonization (Ho et al., 2014; Barbosa and Lery, 2019).

**FIGURE 3:**
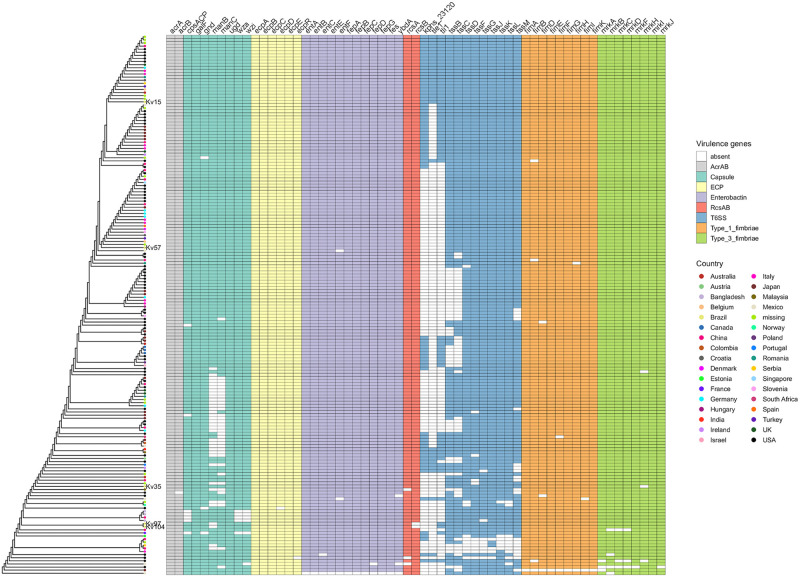
Distribution of *K. pneumoniae* virulence factors among *K. variicola* genomes, represented in a presence/absence matrix of genes. The genomes have been clustered based on their gene content.

For a global perspective, the presence/absence of identified virulence genes was contrasted against several available *K. variicola* genomes. The results show that, apart from genes related to T6SS, the majority of these virulence genes are widespread in the species (Figure 3). The differences observed in this analysis are not sufficient to explain the different phenotypes observed. Moreover, this comparative analysis suggests that these classical *K. pneumoniae* genes are also very conserved in *K. variicola*, indicating that the species may have other genes that contribute to the virulence phenotype as also suggested in previous studies (Potter et al., 2018; Rodríguez-Medina et al., 2019).

### Comparative Genomics

Genome similarity among the five genomes was investigated by calculating their Average Nucleotide Identity (ANI) with fastANI. Aside from an almost identical ANI value (99.99) between strains Kv97 and Kv104, the analysis reveals a minimum ANI of 98.88 between strains Kv35 and Kv104 and a maximum of 99.16 between the strains Kv15 and Kv57. Whole genome homology maps among the five genomes were constructed with Mashmap (Supplementary Figure 1). The resulting dotplots indicate that some of the strains might have regions of duplication (Supplementary Figures 1C,D) but, in general, it shows an overall collinearity between the genomes (Supplementary Figure 1). The homology map between the strains Kv97 and Kv104 shows that their genomes are virtually identical, supporting their high ANI value (Supplementary Figure 1A). These results indicate that the strains Kv97 and Kv104 are clonal, further supported by the inference of only 19 SNPs computed from their whole genome alignment. We extended the comparative genomics analysis by including 296 *K. variicola* complete genomes available in NCBI’s Refseq and creating an all-vs.-all distance matrix based on Dashing software metrics. The projection of this matrix into a two-dimensional space using multidimensional scaling analysis (MDS) enabled the visualization of the relationships of all the genomes (Supplementary Figure 2). This analysis shows that, although isolated from the same hospital, these genomes do not cluster together and individually share genetic relationships to different isolates from the global collection, with the closest genomes to the five strains originating from different countries including the United States, Belgium and China (Table 3). Altogether, this suggests multiple introduction routes and dispersal potential of *K. variicola*.

**TABLE 3:** The closest genome to each of *Klebsiella variicola* strains isolated in Brasilia, DF, Brazil, based on the absolute values of genome distances.

Strain	Closest genome	Genetic distance	Institution	Geographic location
Kv15	GCF_003197065.1	0.006174	Washington university	United States: St. Louis, Missouri
Kv35	GCF_003195165.1	0.006569	Washington university	United States: St. Louis, Missouri
Kv57	GCF_900509865.1	0.004744	Wellcome sanger institute	Belgium
*Kv97 and Kv104	GCF_003285185.1	0.006857	Beijing institute of microbiology and epidemiology	China: Zhejiang

*Based on our results, Kv97 and kv104 seem to be clonal. Thus, we have taken the “second” closest genome to these samples from Dashing results and we could see that the values were identical.

## Discussion

Currently, the misidentification of *K. variicola* as *K. pneumoniae* in routine diagnostics tests is observed. Thus, many worldwide epidemiological data on infections caused by *K. variicola* may have been wrongly attributed to *K. pneumoniae*, underscoring the urge to better characterize this species regarding its genetics, pathogenesis, and ecology, given its ubiquitous distribution (Rodríguez-Medina et al., 2019). In particular, knowledge regarding the genome content of *K. variicola* strains is important to provide insights regarding the plasticity of this species to acquire phenotypes responsible for its adaptation to different niches and hosts, focusing on virulence and AMR determinants.

Here, we first employed three different techniques to identify the five *K. variicola* strains isolated from different patients and sources of infection (Table 1): Vitek2 (BioMérieux), 16S rRNA sequencing, and one-step multiplex PCR protocol (Fonseca et al., 2017). Vitek2 and 16S rRNA sequencing failed to distinguish *K. variicola* from *K. pneumoniae* strains. The difficulty in identifying *K. variicola* by Vitek2 and 16S rRNA sequencing has been pointed out previously by other authors (Rodríguez-Medina et al., 2019). In contrast, the multiplex PCR protocol identified the strains as *K. variicola*, which was later confirmed by sequencing the genome of all strains (Supplementary Table 2).

Based on the results of the antimicrobial susceptibility profile by disk diffusion, all strains were classified as MDR. MDR strains have been reported worldwide including a colistin-resistant hypervirulent strain (Lu et al., 2018). The biological assays showed that the strains presented different capacity to survive and grow under incubation in blood and serum (Figures 1A,B). The strains Kv15, Kv35, and Kv97 presented growth profile in blood, as *K. pneumoniae* virulent strains described by Campos et al. (2018), reaching 1.5–2.5 × 10^9^ CFUs after 60 min of incubation (Figure 1A). Regarding serum survival, all strains presented growth after 60 min of incubation (Figure 1B). Interestingly, Kv15, Kv35, and Kv57 showed ability to invade HEp-2 cells (Figure 1C), as previously observed in virulent *K. pneumoniae* (Campos et al., 2018). These three strains also showed significant higher values of CFUs recovery when compared to Kv97 and Kv104. As these two strains (Kv97 and Kv104) belong to the same clone (Table 1 and Supplementary Table 2), invasion assays showed different pathogenic potential between two groups: (i) one pathogenic group comprised of Kv15, K35, and Kv57 and other (ii), non-pathogenic formed by the clones Kv97 and Kv104. However, additional biological tests as murine infection is recommended to evaluate the virulence potential presented by the strains.

The capsular (K) and lipopolysaccharide (O) serotypes are useful methods for the rapid detection of outbreaks or high-risk clones since individual serotypes often vary in their virulence and invasive potential (Hsieh et al., 2012; Fang et al., 2016; Patro and Rathinavelan, 2019). In this study, we detected four different capsular synthesis loci (KL60, KL71, KL111, and KL20) and one lipopolysaccharide locus shared among the genomes (O3) (Table 2). In *K. variicola*, the prevalence of these serotypes is not yet elucidated and further studies are required. As expected, since they are clonal, the Kv97 and Kv104 strains presented the same K and O antigens, the same ST, and the same genomic content. However, the strains were isolated from different sources and patients: Kv94 from urine and Kv104 from blood, in the same period, January 2017 (Table 1). These results show the emergence of new *K. variicola* clones presenting capacity to colonize and cause infection in different tissues.

In this study, four new *K. variicola* STs (ST168, ST170, ST171, and ST172) have been identified. The investigation of genetic relationships among the MLST profiles deposited at the *K. variicola* database indicates that the *K. variicola* species is separated into two major CGs (Figure 2). Interestingly, this analysis have also divided the strains in two groups as the biological assays have. The pathogenic group (Kv15, Kv35, and Kv57) have been placed in the clonal CG that originates from the ST54, while the non-pathogenic group (Kv97 and Kv104) was placed in the other CG that seems to emerge from ST19 (Figure 2). This observation suggests that these CGs may represent different *K. variicola* populations with different phenotypes and different virulence and resistance dynamics as is known to happen in *K. pneumoniae* (Wyres et al., 2019, 2020). However, more studies are still necessary to better elucidate these differences. Furthermore, the diversity contrast among countries is also highlighted. In Bangladesh, for example, almost all (11/12) the sequenced strains belong to the same ST (ST 60) while in Brazil, almost every genome in the database belongs to a different ST which denotes the necessity of more sequencing efforts. Moreover, the distant genetic relationship among the five strains based on the MLST allelles together with their low ANI values, corroborates with the idea that the diversity of *K. variicola* in Brazil may be greater than what is currently known. In fact, the *K. variicola* MLST database is still new, and the majority of the STs are represented by only one genome. This suggests that the real magnitude of *K. variicola* diversity is yet to be seen and the number of sequenced genomes will be proportional to the contribution to unravel the epidemiology of this species.

Computational searches for ARGs detected those for beta-lactam (*bla*_LEN_), fosfomycin (*fos*A), and quinolone (*oqx*AB). The beta-lactamase genes are one of the most prevalent in the species, being *bla*_LEN_ the most conserved (Potter et al., 2018). This high incidence of chromosomal β-lactamase LEN was expected since *K. variicola* is intrinsically resistant to ampicillin due to the presence of this gene (Rodríguez-Medina et al., 2019). In the same way, the *oqx*AB efflux pump is very conserved in the species as described in a recent study that detected the genes to be widespread among *K. variicola* strains isolated in United States (Potter et al., 2018). In contrast, despite being recently described by Potter et al. (2018) as a not very widespread gene, we identified in this study that virtually all the analyzed *K. variicola* genomes possess the *fos*A gene (Supplementary Material 2), a gene that is chromosomally carried in *K. pneumoniae* (Ito et al., 2017). Altogether these observations indicate that these genes are ubiquitous to the *K. variicola* species and suggest that *K. variicola* strains may be carriers of resistance markers worldwide.

*K. variicola* strains are frequently associated with different types of opportunistic infections. Recently, the first worldwide report of Hmv *K. variicola* causing primary endodontic infection was described in Brazil (Nakamura-Silva et al., 2020). Furthermore, there are studies reporting the convergence of colistin resistance and hypervirulence in *K. variicola* in China (Lu et al., 2018) as well as multi-resistant hypervirulent strains in Bangladesh causing outbreaks with high mortality in newborns (Farzana et al., 2019). The studied strains exhibit genes and phenotypes related to virulence, mirroring factors considered classical *in K. pneumoniae* (Table 2 and Figure 1). These genes were shown to be very conserved among *K. variicola* genomes, except for the T6SS apparatus, and cannot explain the phenotypic differences observed between the strains (Figure 3). One remarkable observation was that although the Hmv phenotype was verified, the transcriptional regulator genes *rmp*A and *mag*A, considered pivotal in *K. pneumoniae*, were not found in the analyzed *K. variicola* strains. Our findings corroborate the results recently described by Nakamura-Silva et al. (2020) and also by Rodríguez-Medina et al. (2019) who did not find these genes in a *K. variicola* Hmv strain. Altogether, these observations corroborate the idea that *K. variicola* employs singular mechanisms of virulence not yet fully elucidated (Rodríguez-Medina et al., 2019).

Overall, our work shows the molecular and genomic characteristics of MDR *K. variicola* strains harboring different virulence factors belonging to novel clones. The results highlighting that *K. variicola* strains corroborating with the possibility that this species has its own virulome that still needs to be further elucidated (Rodríguez-Medina et al., 2019). Furthermore, their plastic genome can be responsible for the acquisitio of determinants that become a potential emerging pathogen.

## Data Availability Statement

The datasets generated in this study are available under the bioproject PRJNA613075 (link: *Klebsiella variicola* (ID 613075)–BioProject–NCBI (nih.gov)).

## Ethics Statement

The studies involving human participants were reviewed and approved by the ethical approval was received from the School of Pharmaceutical Sciences of Ribeirão Preto, University of São Paulo, Brazil (approval no.: CEP/FCFRP 362; CAEE 36031914.9.0000.5403) and from the Faculdade de Medicina, Universidade de Brasília, Brasília, DF, Brazil (approval no. CEP/FMUnB 1.131.054; CAEE: 44867915.1.0000.558). The patients/participants provided their written informed consent to participate in this study.

## Author Contributions

AP-S and TC conceived and designed the experiments. AA and IS carried out biological tests related to bacterial strains isolation, biofilms, human blood and serum survival, bacterial invasion and toxicity to HEp-2 epithelial cell assays, and also statistical analysis. RN-S and MO-S conducted and analyzed the strains identification by sequencing the 16S rRNA and one-step multiplex PCR, molecular typing and MLST, antimicrobial susceptibility, and hypermucosviscous tests. LC and NL carried out the genome sequencing. FA and GP performed genomic and bioinformatics analyzes. All authors contributed to writing this manuscript.

## Conflict of Interest

The authors declare that the research was conducted in the absence of any commercial or financial relationships that could be construed as a potential conflict of interest.

## Footnotes

1 https://github.com/fmalmeida/MpGAP

2 https://github.com/fmalmeida/bacannot

3 https://github.com/tseemann/snippy

4 https://github.com/kblin/ncbi-genome-download

5 http://mlstkv.insp.mx/

## Supplementary Material

Supplementary Figure 1 Whole genome homology maps among the five genomes of this study, constructed with Mashmap. **(A)** Whole genome alignment between Kv97 and Kv104. **(B)** Whole genome alignment between Kv15 and Kv35. **(C)** Whole genome alignment between Kv15 and Kv57. **(D)** Whole genome alignment between Kv15 and Kv97.

Supplementary Figure 2 Projection of the *K. variicola* genome distance matrix estimated by Dashing software into a two dimensional space using multidimensional scaling analysis. The clonal strains Kv97 and Kv104 were projected into the same space, thus only four of the five colored dots are visible.

Supplementary Table 1 Genome assembly statistics for the five *K. variicola* strains analyzed.

Supplementary Material 1 Antimicrobial susceptibilities of the five *Klebsiella variicola* strains and different antimicrobial agents tested.

Supplementary Material 2 The Resfinder detection of ARGs in all the available *K. variicola* genomes. The first sheet summarizes the identification of *fos*A gene among the genomes.

## References

[B1] Alcántar-Curiel M. D. Blackburn D. Saldaña Z. Gayosso-Vásquez C. Iovine N. De La Cruz M. A. (2013). Multi-functional analysis of *Klebsiella pneumoniae* fimbrial types in adherence and biofilm formation. *Virulence* 4:2. 10.4161/viru.22974 23302788 PMC3654611

[B2] Baker D. N. Langmead B. (2019). Dashing: fast and accurate genomic distances with HyperLogLog. *Genome Biol.* 20:265. 10.1186/s13059-019-1875-1870 PMC6892282 31801633

[B3] Barbosa V. A. A. Lery L. M. S. (2019). Insights into *Klebsiella pneumoniae* type VI secretion system transcriptional regulation. *BMC Genomics* 20:506. 10.1186/s12864-019-5885-5889 PMC6580597 31215404

[B4] Barrios-Camacho H. Aguilar-Vera A. Beltran-Rojel M. Aguilar-Vera E. Duran-Bedolla J. Rodriguez-Medina N. (2019). Molecular epidemiology of *Klebsiella variicola* obtained from different sources. *Sci. Rep.* 9:10610. 10.1038/s41598-019-46998-46999 PMC6650414 31337792

[B5] Bortolaia V. Kaas R. S. Ruppe E. Roberts M. C. Schwarz S. Cattoir V. (2020). ResFinder 4.0 for predictions of phenotypes from genotypes. *J. Antimicrobial Chemotherapy* 75 3491–3500. 10.1093/jac/dkaa345 32780112 PMC7662176

[B6] Campos T. A. Gonçalves L. F. Magalhães K. G. Martins V. P. Pappas Júnior G. J. Peirano G. (2018). A fatal bacteremia caused by Hypermucousviscous KPC-2 producing extensively drug-resistant K64-ST11 Klebsiella pneumoniae in Brazil. *Front. Med.* 5:265. 10.3389/fmed.2018.00265 30298131 PMC6161680

[B7] Carattoli A. Zankari E. Garciá-Fernández A. Larsen M. V. Lund O. Villa L. (2014). In Silico detection and typing of plasmids using plasmidfinder and plasmid multilocus sequence typing. *Antimicrobial Agents Chemotherapy* 58 3895–3903. 10.1128/AAC.02412-14 24777092 PMC4068535

[B8] Clinical and Laboratory Standards Institute (CLSI) (2020). *Performance Standards for Antimicrobial Susceptibility Testing, M100*, 30 Edn. Wayne, PA: Clinical and Laboratory Standards Institute (CLSI), M02–M07.

[B9] DeLeo F. R. Kobayashi S. D. Porter A. R. Freedman B. Dorward D. W. Chen L. (2017). Survival of carbapenem-resistant *Klebsiella pneumoniae* sequence type 258 in human blood. *Antimicrobial Agents Chemotherapy* 61:e02533-16. 10.1128/AAC.02533-2516 PMC5365663 28115349

[B10] Fang C. T. Shih Y. J. Cheong C. M. Yi W. C. (2016). Rapid and accurate determination of Lipopolysaccharide O-antigen types in *Klebsiella pneumoniae* with a novel PCR-based O-genotyping method. *J. Clin. Microbiol.* 54 666–675. 10.1128/JCM.02494-15 26719438 PMC4767969

[B11] Farzana R. Jones L. S. Rahman M. A. Andrey D. O. Sands K. Portal E. (2019). Outbreak of hypervirulent multidrug-resistant *Klebsiella variicola* causing high mortality in neonates in Bangladesh. *Clin. Infectious Dis.* 68 1225–1227. 10.1093/cid/ciy778 30204843

[B12] Favre-Bonte S. Joly B. Forestier C. (1999). Consequences of reduction of *Klebsiella pneumoniae* capsule expression on interactions of this bacterium with epithelial cells. *Infect. Immun.* 67 554–561. 9916058 10.1128/iai.67.2.554-561.1999 PMC96354

[B13] Feldgarden M. Brover V. Haft D. H. Prasad A. B. Slotta D. J. Tolstoy I. (2019). Validating the AMRFINder tool and resistance gene database by using antimicrobial resistance genotype-phenotype correlations in a collection of isolates. *Antimicrobial Agents Chemotherapy* 63:e00483-19. 10.1128/AAC.00483-419 PMC6811410 31427293

[B14] Fonseca E. L. Ramos N. da V. Andrade B. G. N. Morais L. L. C. S. Marin M. F. A. (2017). A one-step multiplex PCR to identify *Klebsiella pneumoniae*, *Klebsiella variicola*, and *Klebsiella quasipneumoniae* in the clinical routine. *Diag. Microbiol. Infect. Dis.* 87 315–317. 10.1016/j.diagmicrobio.2017.01.005 28139276

[B15] Gautreau G. Bazin A. Gachet M. Planel R. Burlot L. Dubois M. (2020). PPanGGOLiN: depicting microbial diversity via a partitioned pangenome graph. *PLoS Computat. Biol.* 16:e1007732. 10.1371/journal.pcbi.1007732 32191703 PMC7108747

[B16] Ho B. T. Dong T. G. Mekalanos J. J. (2014). A view to a kill: the bacterial type VI secretion system. *Cell Host Microbe* 15 9–21. 10.1016/j.chom.2013.11.008 24332978 PMC3936019

[B17] Hsieh P. F. Lin T. L. Yang F. L. Wu M. C. Pan Y. J. Wu S. H. (2012). Lipopolysaccharide O1 antigen contributes to the virulence in *Klebsiella pneumoniae* causing pyogenic liver abscess. *PLoS One* 7:e33155. 10.1371/journal.pone.0033155 22427976 PMC3299736

[B18] Ito R. Mustapha M. M. Tomich A. D. Callaghan J. D. McElheny C. L. Mettus R. R. (2017). Widespread fosfomycin resistance in Gram-negative bacteria attributable to the chromosomal fosA gene. *mBio* 8:e00749-17. 10.1128/mBio.00749-717 PMC5574708 28851843

[B19] Jain C. Koren S. Dilthey A. Phillippy A. M. Aluru S. (2018a). A fast adaptive algorithm for computing whole-genome homology maps. *Bioinformatics* 34 i748–i756. 10.1093/bioinformatics/bty597 30423094 PMC6129286

[B20] Jain C. Rodriguez-R L. M. Phyllipy A. M. Konstantinidis K. T. Aluru S. (2018b). High throughput ANI analysis of 90K prokaryotic genomes reveals clear species boundaries. *Nat. Commun.* 9:5114. 10.1038/s41467-018-07641-7649 PMC6269478 30504855

[B21] Jia B. Raphenya A. R. Alcock B. Waglechner N. Guo P. Tsang K. K. (2017). CARD 2017: expansion and model-centric curation of the comprehensive antibiotic resistance database. *Nucleic Acids Res.* 45 D566–D573. 10.1093/nar/gkw1004 27789705 PMC5210516

[B22] Liu B. Zheng D. Jin Q. Chen L. Yang J. (2019). VFDB 2019: a comparative pathogenomic platform with an interactive web interface. *Nucleic Acids Res.* 47 D687–D692. 10.1093/nar/gky1080 30395255 PMC6324032

[B23] Lu Y. Feng Y. McNally A. Zong Z. (2018). Occurrence of colistin-resistant hypervirulent *Klebsiella variicola*. *J. Antimicrobial Chemotherapy* 73 3001–3004. 10.1093/jac/dky301 30060219

[B24] Maatallah M. Vading M. Kabir M. H. Bakhrouf A. Kalin M. Nauclér P. (2014). Klebsiella variicola is a frequent cause of bloodstream infection in the Stockholm area, and associated with higher mortality compared to *K. pneumoniae*. *PLoS One* 9:e113539. 10.1371/journal.pone.0113539 25426853 PMC4245126

[B25] Magiorakos A. P. Srinivasan A. Carey R. B. Carmeli Y. Falagas M. E. Giske C. G. (2012). Multidrug-resistant, extensively drug-resistant and pandrug-resistant bacteria: an international expert proposal for interim standard definitions for acquired resistance. *Clin. Microbiol. Infect.* 18 268–281. 10.1111/j.1469-0691.2011.03570.x 21793988

[B26] Mazloum M. le Meur M. Barnaud G. Messika J. (2016). Hypermucoviscous *Klebsiella pneumoniae* pneumonia: follow the string! *Intensive Care Med.* 42 2092–2093. 10.1007/s00134-016-4363-y 27139020

[B27] Meier-Kolthoff J. P. Göker M. (2019). TYGS is an automated high-throughput platform for state-of-the-art genome-based taxonomy. *Nat. Commun.* 10:2182. 10.1038/s41467-019-10210-10213 PMC6522516 31097708

[B28] Nakamura-Silva R. Martins Domingues, de Macedo L. Teixeira L. Oliveira, da Silva M. (2020). First report of hypermucoviscous *Klebsiella variicola* subsp. variicola 2 causing primary endodontic infection. *Clin. Microbiol. Infect.* 27 303–304. 32771643 10.1016/j.cmi.2020.07.045

[B29] Patro L. P. P. Rathinavelan T. (2019). Targeting the sugary armor of *Klebsiella* species. *Front. Cell Infect. Microbiol.* 9:367. 10.3389/fcimb.2019.00367 31781512 PMC6856556

[B30] Piepenbrock E. Higgins P. G. Wille J. Xanthopoulou K. Zweigner J. Jahn P. (2020). Klebsiella variicola causing nosocomial transmission among neonates – an emerging pathogen? *J. Med. Microbiol.* 69 396–401. 10.1099/jmm.0.001143 32125266

[B31] Potter R. F. Lainhart W. Twentyman J. Wallace M. A. Wang B. Burnham C. A. D. (2018). Population structure, antibiotic resistance, and uropathogenicity of *Klebsiella variicola*. *mBio* 9:e02481-18. 10.1128/mBio.02481-2418 PMC6299229 30563902

[B32] Rodríguez-Medina N. Barrios-Camacho H. Duran-Bedolla J. Garza-Ramos U. (2019). *Klebsiella variicola*: an emerging pathogen in humans. *Emerg. Microbes Infect.* 8 973–988. 10.1080/22221751.2019.1634981 31259664 PMC6609320

[B33] Seppey M. Manni M. Zdobnov E. M. (2019). BUSCO: assessing genome assembly and annotation completeness. *Methods Mol. Biol.* 1962 227–245. 10.1007/978-1-4939-9173-0_14 31020564

[B34] Srinivasan V. B. Rajamohan G. (2020). Comparative genome analysis and characterization of a MDR *Klebsiella variicola*. *Genomics* 112 3179–3190. 10.1016/j.ygeno.2020.06.004 32504650

[B35] Stepanović S. Ćirković I. Ranin L. Švabić-Vlahović M. (2004). Biofilm formation by *Salmonella* spp. and Listeria monocytogenes on plastic surface. *Lett. Appl. Microbiol.* 38 428–432. 10.1111/j.1472-765X.2004.01513.x 15059216

[B36] Swick M. C. Morgan-Linnell S. K. Carlson K. M. Zechiedrich L. (2011). Expression of multidrug efflux pump genes acrAB-tolC, mdfA, and norE in *Escherichia coli* clinical isolates as a function of fluoroquinolone and multidrug resistance. *Antimicrobial Agents Chemotherapy* 55 921–924. 10.1128/AAC.00996-910 21098250 PMC3028778

[B37] Weisburg W. G. Barns S. M. Pelletier D. A. Lane D. J. (1991). 6S ribosomal DNA amplification for phylogenetic study. *J Bacteriol.* 173 697–703. 1987160 10.1128/jb.173.2.697-703.1991 PMC207061

[B38] Wick R. R. Heinz E. Holt K. E. Wyres K. L. (2018). Kaptive web: user-friendly capsule and lipopolysaccharide serotype prediction for *Klebsiella* genomes. *J. Clin. Microbiol.* 56:e00197-18. 10.1128/JCM.00197-118 PMC5971559 29618504

[B39] Wick R. R. Judd L. M. Gorrie C. L. Holt K. E. (2017). Unicycler: resolving bacterial genome assemblies from short and long sequencing reads. *PLOS Comput. Biol.* 13:e1005595. 10.1371/journal.pcbi.1005595 28594827 PMC5481147

[B40] Wilksch J. J. Yang J. Clements A. Gabbe J. L. Short K. R. Cao H. (2011). MrKH, a novel c-di-GMP-dependent transcriptional activator, controls *klebsiella pneumoniae* biofilm formation by regulating type 3 fimbriae expression. *PLoS Pathogens* 7:e1002204. 10.1371/journal.ppat.1002204 21901098 PMC3161979

[B41] Wyres K. L. Lam M. M. C. Holt K. E. (2020). Population genomics of *Klebsiella pneumoniae*. *Nat. Rev. Microbiol.* 18 344–359. 10.1038/s41579-019-0315-311 32055025

[B42] Wyres K. L. Wick R. R. Judd L. M. Froumine R. Tokolyi A. Gorrie C. L. (2019). Distinct evolutionary dynamics of horizontal gene transfer in drug resistant and virulent clones of *Klebsiella pneumoniae*. *PLoS Genetics* 15:e1008114. 10.1371/journal.pgen.1008114 30986243 PMC6483277

[B43] Zhou Z. Alikhan N. F. Sergeant M. J. Luhmann N. Vaz C. Francisco A. P. (2018). Grapetree: visualization of core genomic relationships among 100,000 bacterial pathogens. *Genome Res.* 28 1395–1404. 10.1101/gr.232397.117 30049790 PMC6120633

