# Supplementary figures and images for: Multidrug-Resistant (MDR) Klebsiella variicola Strains Isolated in a Brazilian Hospital Belong to New Clones
Source: Front Microbiol. 2021 Apr 16;12:604031. doi: 10.3389/fmicb.2021.604031 (PMC8085564; doi:10.3389/fmicb.2021.604031)

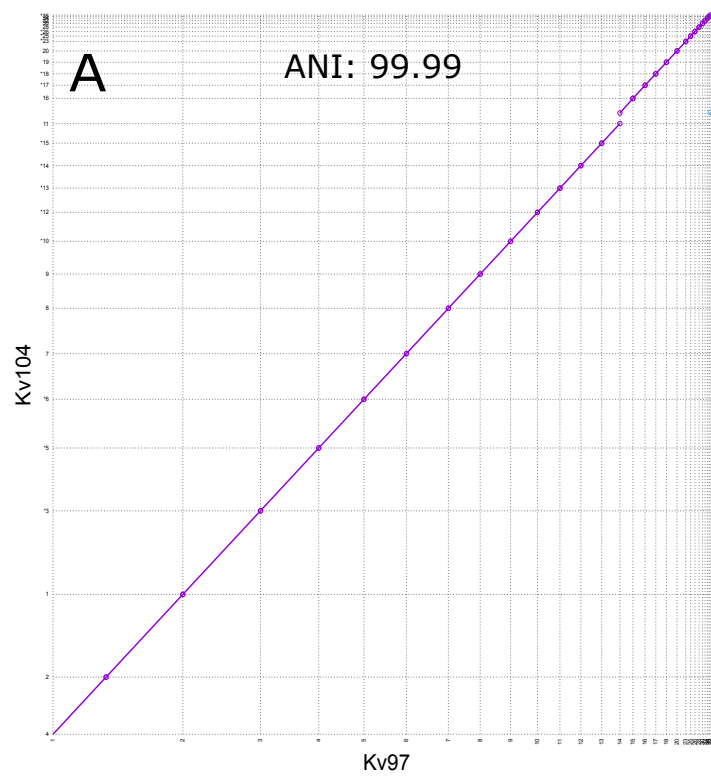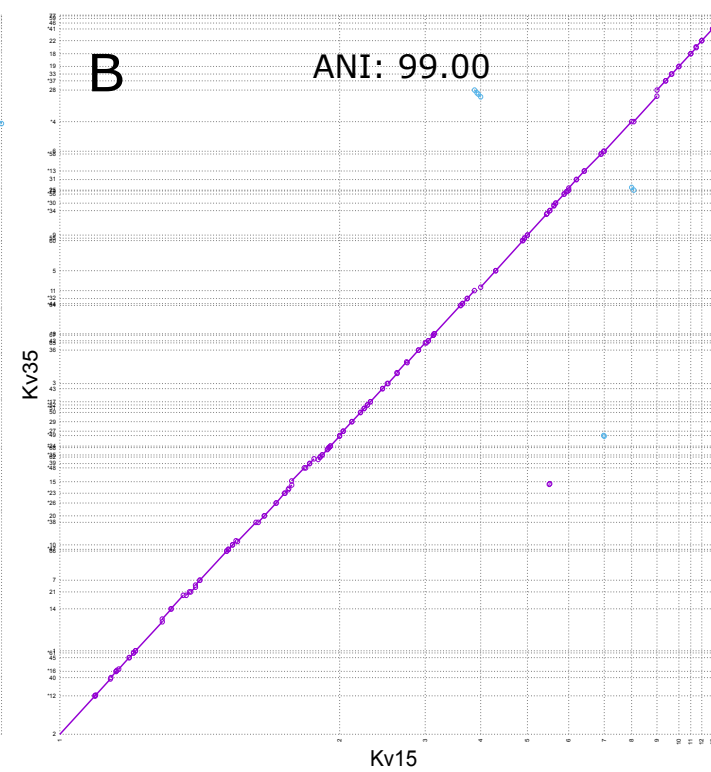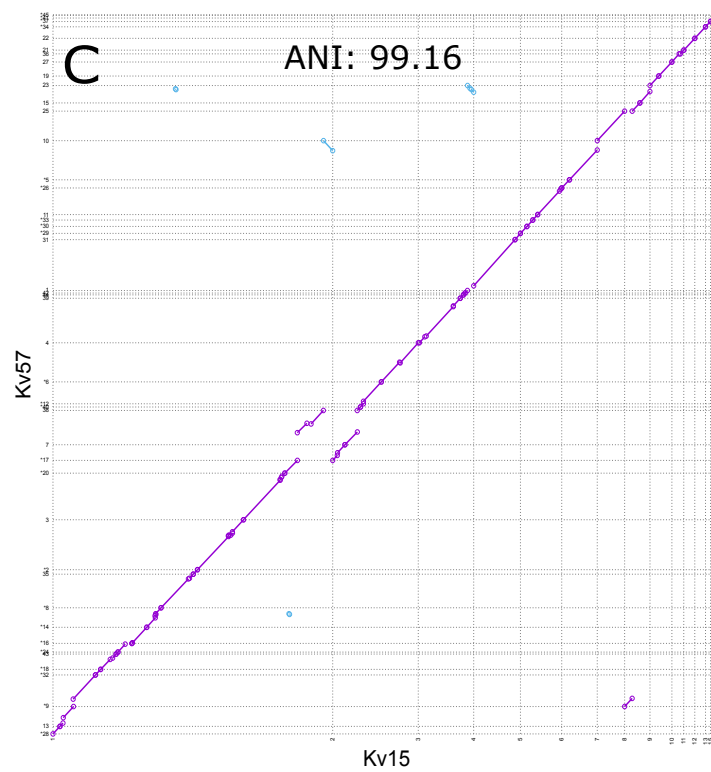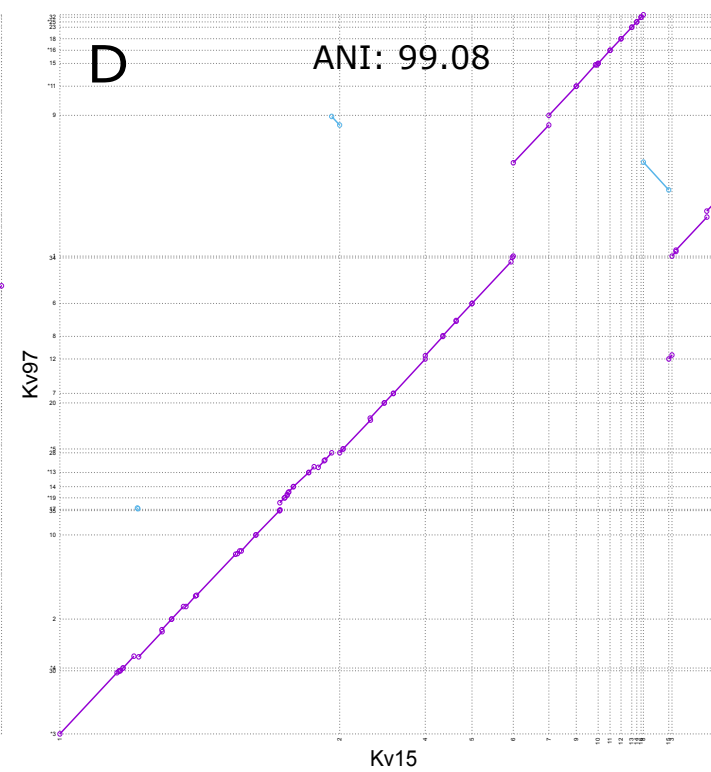

Supplement: Supplementary Figure 1 — Whole genome homology maps among the five genomes of this study, constructed with Mashmap. (A) Whole genome alignment between Kv97 and Kv104. (B) Whole genome alignment between Kv15 and Kv35. (C) Whole genome alignment between Kv15 and Kv57. (D) Whole genome alignment between Kv15 and Kv97. [file Image_1.pdf]

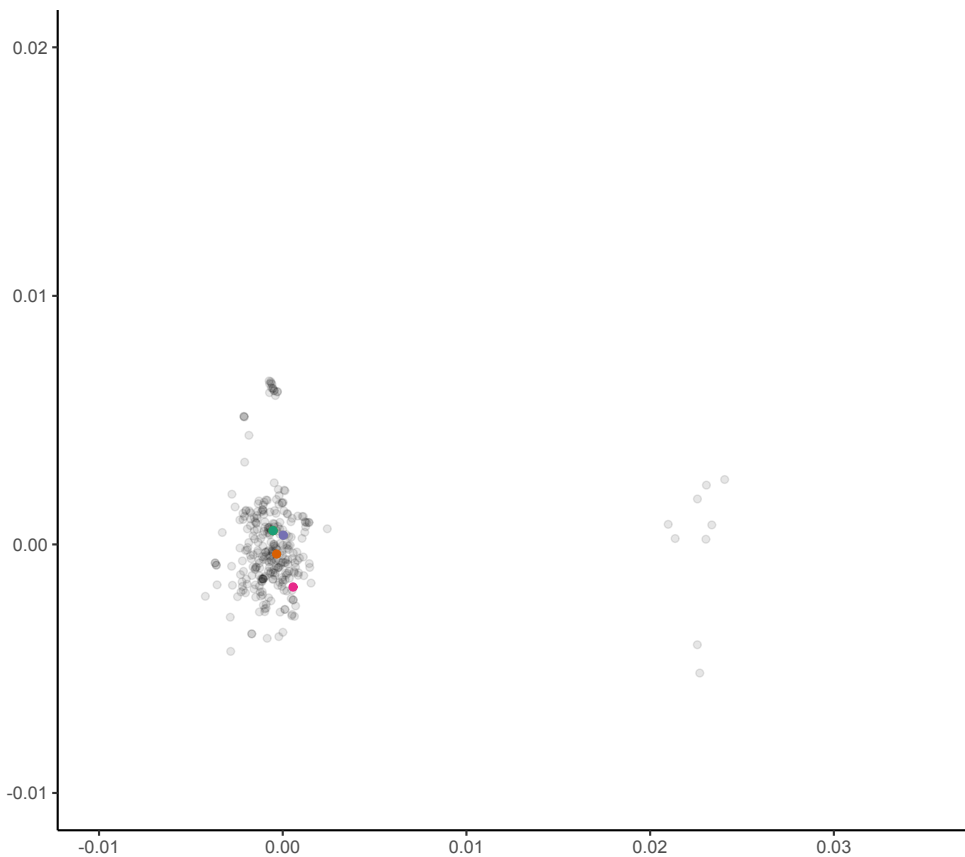

Samples    ● Kv104    ● Kv15    ● Kv35    ● Kv57    ● Kv97

Supplement: Supplementary Figure 2 — Projection of the K. variicola genome distance matrix estimated by Dashing software into a two dimensional space using multidimensional scaling analysis. The clonal strains Kv97 and Kv104 were projected into the same space, thus only four of the five colored dots are visible. [file Image_2.pdf]
