# Supplementary material for: Multidrug-Resistant (MDR) Klebsiella variicola Strains Isolated in a Brazilian Hospital Belong to New Clones
Source: Front Microbiol. 2021 Apr 16;12:604031. doi: 10.3389/fmicb.2021.604031 (PMC8085564; doi:10.3389/fmicb.2021.604031)
Supplement: Supplementary Table 1 — Genome assembly statistics for the five K. variicola strains analyzed. [file Table_1.docx]

Supplementary Table 1. Genome assembly statistics for the five *K. variicola* strains analyzed

| Strain | Genome accession (NCBI) | Estimated short read Coverage | Assembly size (Mb) | Contigs (>=200 bp) | N50 | BUSCO completeness (%) | Plasmid replicons |
| --- | --- | --- | --- | --- | --- | --- | --- |
| Kv15 | GCA_012274425.1 | 70x | 5.4 | 33 | 689,161 | 98.6 |  |
| Kv35 | GCA_012274365.1 | 45x | 5.5 | 125 | 125,327 | 97.9 | IncFIB(K) |
| Kv57 | GCA_012274355.1 | 35x | 5.4 | 49 | 235,608 | 97.5 |  |
| Kv97 | GCA_012274385.1 | 70x | 5.7 | 72 | 347,854 | 98.6 | IncFIB(K) |
| Kv104 | GCA_012274345.1 | 70x | 5.7 | 72 | 347,853 | 98.6 | IncFIB(K) |
